# Genetic Links Between Subcortical Brain Morphometry and Suicide Attempt Risk in Children and Adults

**DOI:** 10.1002/hbm.70220

**Published:** 2025-05-13

**Authors:** Zuriel Ceja, Luis M. García‐Marín, I‐Tzu Hung, Sarah E. Medland, Alexis C. Edwards, Miguel E. Rentería, Jill A. Rabinowitz

**Affiliations:** ^1^ Brain & Mental Health Program QIMR Berghofer Medical Research Institute Brisbane Australia; ^2^ School of Biomedical Sciences Faculty of Health, Medicine and Behavioural Sciences, the University of Queensland Brisbane Australia; ^3^ Department of Psychiatry Robert Wood Johnson Medical School, Rutgers University Piscataway USA; ^4^ Department of Psychiatry Virginia Institute for Psychiatric and Behavioral Genetics, Virginia Commonwealth University Virginia USA; ^5^ School of Biomedical Sciences Faculty of Health, Queensland University of Technology Brisbane Australia

**Keywords:** brain morphometry, intracranial volume, subcortical brain structures, suicidality, suicide attempt

## Abstract

Genome‐wide association studies (GWAS) have uncovered genetic variants associated with suicide attempt (SA) risk and regional brain volumes (RBVs). However, the extent of their genetic overlap remains unclear. To address this, we investigated whether the genetic architecture of SA and various RBVs (i.e., caudate nucleus, hippocampus, brainstem, ventral diencephalon, thalamus, globus pallidus, putamen, nucleus accumbens, amygdala and intracranial volume (ICV)) was shared. We leveraged GWAS summary statistics from the largest available datasets on SA (*N* = 958,896) and intracranial and subcortical RBVs (*N* = 74,898). Using linkage disequilibrium score regression, we estimated genome‐wide genetic correlations between SA and individual RBVs. GWAS‐pairwise analyses identified genomic segments associated with both SA and RBVs, followed by functional annotation. Additionally, we examined whether polygenic scores (PGS) for SA were associated with ICV and subcortical brain structure phenotypes in youth of European ancestry (*N* = 5276) in the Adolescent Brain Cognitive Development (ABCD) study. Linkage disequilibrium score regression results indicated a significant genetic correlation between SA and ICV (rG = −0.10, *p*‐value = 1.9 × 10–3). GWAS‐pairwise analyses and functional annotation revealed 10 genomic segments associated with SA and at least one RBV (thalamus, putamen and caudate nucleus). After adjusting for multiple tests, PGS association analysis indicated that a higher PGS for SA was significantly associated with a smaller volume of the right nucleus accumbens (*b* = −7.05, *p* = 0.018). Our findings highlight a negative genetic correlation between SA and ICV amongst adults and suggest different neural correlates associated with genetic risk for SA across developmental periods. This study advances our understanding of the shared genetic underpinnings of SA and brain structure, potentially informing future research and clinical interventions.

## Graphical abstract

Our findings indicate that genetic variation in brain morphometry and suicide attempt is shared across the lifespan.

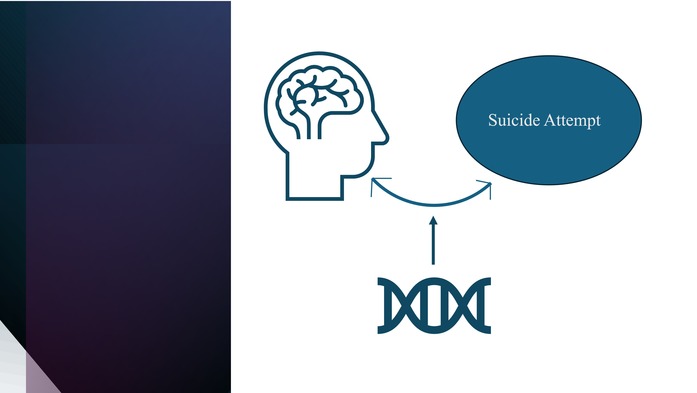

## 1 Introduction

Suicide attempt (SA) is a significant global public health issue with a substantial contribution to morbidity and mortality worldwide (World Health Organisation 2023). Recent large‐scale neuroimaging and genetic studies have enabled the identification of neurobiological areas and genetic variants associated with suicidal behaviours, such as SA (Docherty et al. 2023; Campos et al. 2021; van Velzen et al. 2022; Ceja et al. 2025). However, the extent to which genetic factors contribute to both SA and variation in brain structure remains unclear, leaving a critical gap in our understanding of potential shared biological pathways. Investigating this overlap is essential for elucidating the underlying biological mechanisms and identifying novel biomarkers and therapeutic targets for prevention and intervention strategies.

### 1.1 Phenotypic Links Between Brain Structure and SA

At the phenotypic level, numerous studies have linked brain structure alterations with SA (van Velzen et al. 2022; Campos et al. 2021). Evidence suggests that these associations may differ across developmental periods. For example, in adults, a smaller left inferior parietal lobe surface area and reduced volumes in the right pallidum and the left and right thalamus have been associated with lifetime SA (Campos et al. 2021). Similarly, in a subsample of middle to late adolescents with mood disorders, individuals with a history of SA had a smaller surface area of the frontal pole compared to those without a lifetime history of SA (van Velzen et al. 2022). Additionally, a study identified differences in surface area in the prefrontal, temporal and parietal regions in adolescent patients with a history of SA compared with healthy controls (Gifuni et al. 2021). These findings underscore the potential of brain morphological characteristics as markers of SA risk and highlight the necessity for exploring their genetic underpinnings across different developmental stages.

### 1.2 Genetics of Neural Structures and SA

Recent genome‐wide association studies (GWAS) and genetic enrichment analyses support a genetic basis for SA within specific brain regions. For example, a recent GWAS (*N* = 958,896) identified 12 loci associated with SA (*p* value < 5 × 10^−8^), indicating that SA is moderately heritable. The study reported a SNP‐based heritability for SA (ℎ^2^_snp_ = 5.7%, SE = 0.003, *p* = 5.7 × 10^−80^) and found significant genetic enrichment in multiple brain regions and other tissues (Docherty et al. 2023). The strongest enrichment was found in genes expressed in the pituitary gland (*b* = 0.035601, *p* = 6.3E‐05), highlighting its potential involvement in the aetiology of SA. The other structures showing significant enrichment included the cerebellar hemisphere and cerebellum, frontal cortex BA9, anterior cingulate cortex, amygdala, nucleus accumbens, hippocampus, hypothalamus, caudate nucleus and putamen.

Brain morphometry is also heritable, with genetics explaining up to 80% of individual variation in the brain structure (median heritability 34.8%) (Roshchupkin et al. 2016; Zhao et al. 2019). For instance, the regional brain volumes (RBVs) with the largest SNP‐based heritability estimates include the brainstem (82.7%), cerebellar vermal lobules VIII.X (68.3%), cerebellar vermal lobules I.V (68.0%), brain volume (65.9%) and left cerebellum exterior (64.1%) (Zhao et al. 2019). These genetic findings suggest a complex interplay between genetics and neurobiology (Mullins et al. 2022). Despite these findings, it remains unclear whether specific genetic loci are shared between SA and subcortical RBVs, leaving a critical gap in understanding the genetic correlation between these traits. Such work may allow for a deeper understanding of the biological pathways involved in SA and aid in identifying novel biomarkers implicated in SA risk.

### 1.3 Current Study

We sought to build on previous research by identifying shared genetic factors and specific genomic segments between SA and RBVs. Previous studies suggest that specific genes may be intricately linked to suicidal behaviors; however, findings have been limited by small sample sizes, contributing to inconsistent and underpowered results (Willour et al. 2012; Mullins et al. 2014). To address these limitations, we utilised the largest and most well‐powered SA GWAS (Docherty et al. 2023) to examine the genetic overlap between SA and RBVs comprehensively.

First, we conducted both genome‐wide and local correlation analyses to identify shared genetic aetiology between SA and RBVs. Specifically, we employed linkage disequilibrium score regression (LDSC) (Bulik‐Sullivan et al. 2015) to estimate genome‐wide correlations between SA and intracranial and nine subcortical RBVs. Second, we used GWAS‐pairwise (GWAS‐PW) (Pickrell et al. 2016) and functional annotation (Watanabe et al. 2017) to further elucidate the genetic architecture underlying these traits. This approach allowed us to determine shared genetic factors, thus enhancing our understanding of the genetic basis of SA and its relationship with specific RBVs. Third, we constructed polygenic scores (PGS) (weighted aggregate scores reflecting an individual's genetic liability) for SA and examined their associations with phenotypic RBVs in the Adolescent Brain Cognitive Development (ABCD) study. This analysis aimed to identify whether genetic predisposition to SA is associated with structural variations in the brain during adolescence.

## 2 Methods

### 2.1 Datasets

#### 2.1.1 Suicide Attempt GWAS Data

We leveraged GWAS summary statistics from a meta‐analysis in individuals of European ancestry, including 35,786 SA cases and 779,392 controls (Docherty et al. 2023). SA cases were defined as nonfatal SA s (self‐injurious behaviour with clear intent to die) and suicide deaths. Specifically, the European GWAS combined data from 15 cohorts: 13 cohorts with SA only, one cohort with suicide death only and one cohort that included both SA and suicide death cases. An inverse variance‐weighted fixed effects model was used to implement the meta‐analysis in METAL (Willer et al. 2010). Participants included individuals of European ancestry from The International Suicide Genetics Consortium (ISGC; now renamed to Psychiatric Genomics Consortium Suicide Working Group) (Mullins et al. 2022) and the Million Veteran Program (MVP) (Kimbrel et al. 2022). A detailed description of these GWAS summary statistics is available elsewhere (Docherty et al. 2023) and was obtained via direct application and data transfer agreement approval.

#### 2.1.2 Intracranial and Subcortical Brain Volumes GWAS Data

We used GWAS summary statistics for intracranial and nine subcortical RBVs, including those of the caudate nucleus, hippocampus, brainstem, ventral diencephalon, thalamus, globus pallidus, putamen, nucleus accumbens and amygdala (García‐Marín et al. 2024). Briefly, GWAS meta‐analyses were performed using MTAG (Turley et al. 2018) by leveraging magnetic resonance imaging (MRI) data in up to 74,898 individuals of European ancestry from several international datasets, including the UK Biobank (Elliott et al. 2018) and ABCD (Feldstein Ewing et al. 2018) cohorts and the ENIGMA (Medland et al. 2022) and CHARGE (Thompson 2015) consortia. Intracranial and subcortical RBVs included the mean volume of both hemispheres, measured in cubic centimetres, except for the brainstem, for which the total volume was used (García‐Marín et al. 2024). Further details regarding phenotype definition and ENIGMA's protocols are available elsewhere (Satizabal et al. 2019; Thompson et al. 2014, 2020). GWAS for the UK Biobank and ABCD cohorts were conducted with a linear mixed‐effects model using BOLT‐LMM (v2.3.2) (García‐Marín et al. 2024). Covariates included genotyping array, sex, age, sex*age, age^2^, sex*age^2^ and the first 20 genetic principal components (PCs) (García‐Marín et al. 2024). The RVBs of the nine subcortical brain structures used in the present study were adjusted for total intracranial volume (ICV), and genetic variants with a low‐quality imputation score (< 0.60) or a low minor allele frequency (< 0.01) were excluded (García‐Marín et al. 2024).

#### 2.1.3 Adolescent Brain Cognitive Development Study

##### 2.1.3.1 Participants

ABCD is a population‐based study aimed at understanding environmental, biobehavioral, neural and genetic predictors of substance use across adolescence. Children (*N* = 11,868) aged 9–10 years and their caregivers were recruited at 21 sites across the US. The analytic sample (*N* = 5267) was limited to individuals who had valid genetic data, were identified as European through genetic ancestry analysis and had completed a neuroimaging assessment at baseline.

##### 2.1.3.2 Phenotypic Intracranial and Subcortical Brain Volumes

We examined ICV and RBVs of nine subcortical structures (caudate nucleus, hippocampus, brainstem, ventral diencephalon, thalamus, globus pallidus, putamen, nucleus accumbens and amygdala) at baseline when children were approximately 9–10 years of age. Data drawn from the curated annual data release 5.1. Across the 21 ABCD data collection sites, MRI protocols were harmonised to facilitate analysis. ABCD used 3‐T scanners using either 32‐channel head or 64‐channel head/neck coils (based on availability) and included 3D isovolumetric T1‐ and T2‐weighted sequences. When available, MRI scans included prospective motion correction. Central processing and analysis of the MRI data was conducted by the ABCD Data Analysis and Informatics Center (DAIC). Researchers used Multi‐Modal Processing Stream (MMPS); this is a software package that was developed and is housed at the Center for Multimodal Imaging and Genetics (CMIG) at the University of California, San Diego (UCSD), which enabled the analysis of large‐scale, multimodal neuroimaging data analysis. Numerous automated and manual pre‐processing and quality control procedures were conducted, with detailed processing steps documented here (https://www.nitrc.org/projects/abcd_study). More detailed information regarding the MRI protocols can be found elsewhere (Casey et al. 2018; Hagler Jr et al. 2019).

## 3 Statistical Analyses

### 3.1 Genetic Correlation Estimation

We used LDSC to estimate genome‐wide genetic correlations between SA and intracranial and subcortical RBVs (Bulik‐Sullivan et al. 2015). We applied Bonferroni multiple testing correction to determine statistical significance (i.e., 0.05/10 [number of brain structures] = 0.005).

### 3.2 GWAS‐Pairwise

We used the GWAS‐PW method to delineate specific genomic segments influencing the aetiology of SA and the volume of at least one RVB as described in previous studies (Mitchell et al. 2022; García‐Marín et al. 2023; Diaz‐Torres et al. 2023). Specifically, we performed separate GWAS‐PW analyses for SA and each of the nine subcortical RVBs and ICV.

The GWAS‐PW method splits the genome into 1703 independent genomic segments based on LD patterns. We estimated the posterior probability of association (PPA) for each genomic segment for four models. These included (i) the genomic segment is only associated with SA, (ii) the genomic segment is only associated with a specific RVB, (iii) the genomic segment influences the aetiology of both traits via the same causal genetic variants and (iv) the genomic segment influences the aetiology of both traits via different genetic variants (Mitchell et al. 2020; García‐Marín et al. 2023). In the present study, we present findings for segments of the genome where model three showed a PPA > 0.5, as this threshold has been used in previous studies (Mitchell et al. 2020; García‐Marín et al. 2023).

### 3.3 Functional Annotation

We performed gene‐based test analyses at the genome‐wide level using the FUMA online platform (v1.3.7) (Watanabe et al. 2017). We also extracted findings for the specific genome segments identified via the GWAS‐PW method. FUMA implements MAGMA (de Leeuw et al. 2015) (v1.08) and conducts gene‐based association analyses. We report associations for genes after applying a Bonferroni multiple testing correction defined as *p* = 0.05 / [total number of genes for each subcortical RBV].

### 3.4 Conditional and Linkage Disequilibrium Analyses

#### 3.4.1 Conditional Analysis Using GCTA‐COJO

To investigate whether the seven shared genomic segments between SA and thalamus RBV within the major histocompatibility complex (MHC) region represent independent genetic signals or a single shared variant influenced by the region's complex LD structure, we conducted a conditional and joint association analysis (COJO) using GCTA software (v1.93.2) (Yang et al. 2021). We specifically focused on the thalamus because the shared genomic segments associated with its RBV were located on the short arm of chromosome 6 within the MHC region. This region contains the most polymorphic gene cluster in the human genome, making it particularly challenging to disentangle overlapping genetic signals.

We used genotype data from individuals of European ancestry in the 1000 Genomes Project Phase 3 as the LD reference panel for the COJO analysis. The reference panel provides estimates of LD patterns needed for the conditional analysis. We performed stepwise model selection in COJO to identify independent lead SNPs within the MHC region for each trait. We then conducted conditional analyses by conditioning the association of SA on the lead SNP identified for thalamus RBV and vice versa.

#### 3.4.2 Linkage Disequilibrium Analysis Using PLINK

We calculated pairwise LD between the lead SNPs identified in the COJO analysis using PLINK v1.9 (Purcell et al. 2007). We computed the squared correlation coefficient (*R* ^2^) and D' between the lead SNPs rs34816374 (SA) and rs9295687 (thalamus RVB) using the same LD reference panel from the 1000 Genomes Project. The LD was calculated using the –ld and –r2 options in PLINK, which provide measures of LD between specified SNP pairs. Focusing on the lead SNPs allowed us to examine whether the observed associations were driven by complex linkage disequilibrium within the MHC region, which could result in multiple signals originating from a single genetic source.

### 3.5 PGS Estimation and Analyses in ABCD

Based on the largest GWAS to date on SA (Docherty et al. 2023), we created PGS for SA in the ABCD sample. The ABCD sample was not included in the GWAS under study, and thus, sample dependence was not a concern. A Bayesian method, sBayesR, was used to calculate the PGS (Lloyd‐Jones et al. 2019) implemented within the Genome‐wide Complex Trait Bayesian analysis (GCTB v2.0) software tool (Chung 2021). Relative to other approaches, SBayesR can reduce potential biases due to SNP correlations in linkage disequilibrium by estimating joint effect sizes (Lloyd‐Jones et al. 2019). Quality control measures consisted of removing SNPs with low imputation quality (*r*2 < 0.06), call rate > 0.9, non‐autosomal and strand ambiguous SNPs and SNPs with a low minor allele frequency (> 0.01). To determine the association of SA PGS with ICV and RBVs of subcortical structures, we used neuroimaging data drawn from ABCD Study Release 5.1, which included measurements of ICV and RBVs for structures in both the right and left hemispheres.

We conducted linear mixed effects models in R Version 4.3.1 using the *nlme* package. Across models, age, age^2^, sex, age*sex and age*sex^2^ were included as covariates in the analyses; however, in models that included subcortical structures, we additionally adjusted for ICV. To account for population stratification, we also included the first 10 genetic PCs as covariates. In PGS analyses, we adjusted for multiple testing by applying the Benjamini‐Hochberg^59^ method (false discovery rated‐adjusted *p* value ≤ 0.05). We present both unadjusted and FDR‐adjusted *p* values in the results text and tables. Family ID was included as a random effect to account for family clustering.

## 4 Results

### 4.1 Genome‐Wide and Local Genetic Overlap

**FIGURE 1:**
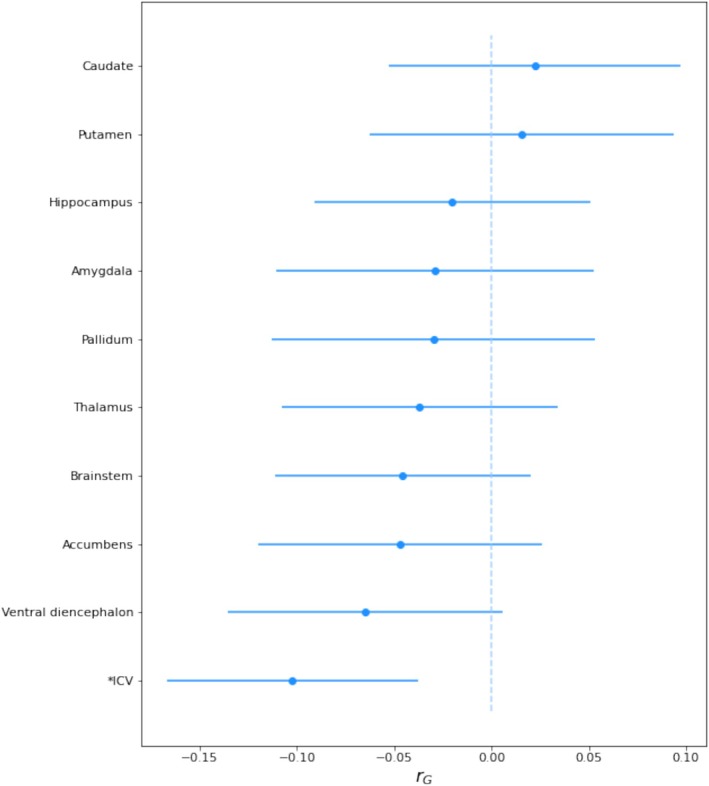
LDSC estimates of the genetic correlation (rG) with 95% confidence intervals between suicide attempt and the volume of nine subcortical brain structures adjusted for intracranial volume. Statistically significant genetic correlations (i.e., *p* < 0.05/10 [number of brain structures]) are marked with an asterisk (*). ICV = intracranial volume.

Using LDSC, we investigated the genetic correlation at the genome‐wide level between SA and 10 RBV structures (Figure 1). After correcting for multiple testing, we identified one genetic correlation that was statistically significant: SA and ICV (rG = −0.10, *p*‐value = 1.9 × 10^−3^). We further investigated the relationship between SA and nine subcortical RBVs and ICV at the local genetic level using GWAS‐PW. Through this approach, we identified 10 genomic segments with genetic variants involved in the aetiology of SA and at least one RBV. The RBV of the thalamus showed the largest number of genomic segments (*N* = 7) with SA, followed by the RBVs of the putamen (2 segments) and caudate nucleus (1 segment).

### 4.2 Functional Annotation

**FIGURE 2:**
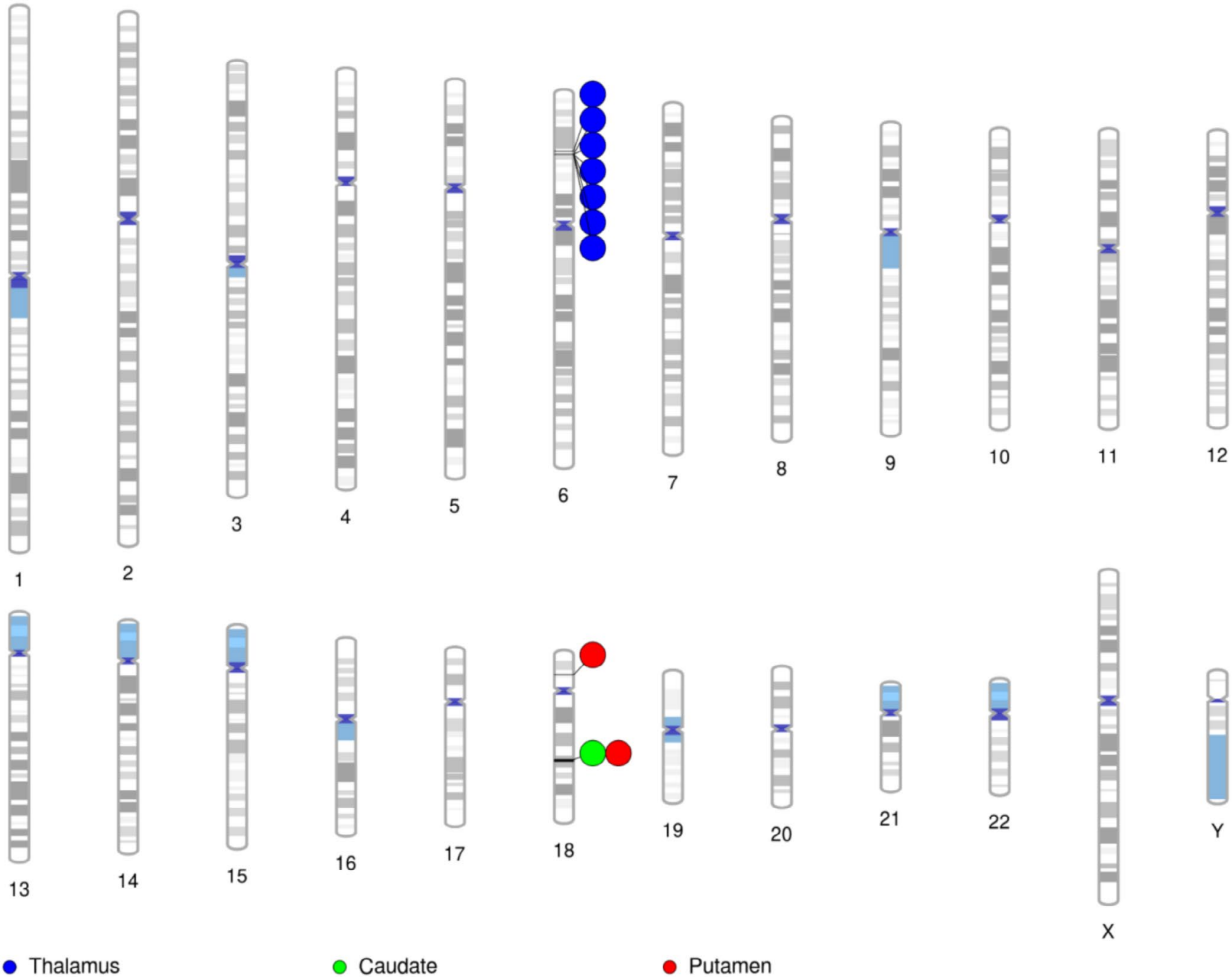
Ideogram highlighting genes associated with brain structures and suicide attempt.

For each RBV with shared genomic segments with SA, we mapped genetic variants in the identified genomic segments to protein‐coding genes using MAGMA (de Leeuw et al. 2015) (v1.08) (Table 1). Nine genes were statistically significantly associated with SA and at least one RBV (Figure 2). In the present study, the RBV of the thalamus and SA were associated with seven genes, including *BTN3A2*, *HIST1H1B*, *HIST1H2BL*, *HIST1H2BN*, *HIST1H2AJ*, *HIST1H4L* and *OR2B2*. The RVBs of the putamen and caudate nucleus were associated with two (*PPP4R1* and *DCC*) and one (*DCC*) gene also associated with SA, respectively.

**TABLE 1:** Genes in genomic segments jointly influencing brain morphometry and suicide attempt.

Gene associated with suicide attempt	Brain structures associated with the gene	Biological relevance of the gene
*BTN3A2*	Thalamus	Associated with Major Depressive Disorder (MDD) (Yang et al. 2021) and schizophrenia in the DL PFC (Li et al. 2022a, 2022b)
*HIST1H1B*	Thalamus	It can generate superoxide and inactivate enzymes from oxidative defence by unfavourably participating in the host defence of *Streptococcus pneumoniae* pneumonia (Liu et al. 2018).
*HIST1H2BL*	Thalamus	It is associated with metabolism and molecular decreased activity or degeneration (Bansal et al. 2021).
*HIST1H2BN*	Thalamus	Linked to schizophrenia, depression, bipolar disorder and autism spectrum disorder(Yulu Wu et al. 2020).
*HIST1H2AJ*	Thalamus	Contributes to chronic inflammation and ageing‐associated diseases (Bansal et al. 2021).
*HIST1H4L*	Thalamus	It is involved in DNA binding and has an effect on gene regulation (Yulu Wu et al. 2020).
*OR2B2*	Thalamus	Linked to Alzheimer's disease in ethnicity‐specific population data (Shigemizu et al. 2021).
*DCC*	Putamen Caudate nucleus	It influences miR‐218 in the prefrontal cortex of MDD, and reduced expression of this gene in the prefrontal cortex neurons was related to elevated resilience against stress‐induced depression (Roy and Dwivedi 2018; Torres‐Berrío et al. 2017).
*PPP4R1*	Putamen	It has been reported to be part of tumour progression, especially having an accelerated growth effect in hepatocellular carcinoma and breast cancer cells (Zhu et al. 2016).

Notably, all seven genes associated with both SA and thalamus RVB are located within a relatively narrow region on chromosome 6, specifically within the MHC region. The top SNPs from each GWAS in this region are rs35869525 (chr6:26,946,687 bp) for thalamus RBV and rs9295687 (chr6:26,082,710 bp) for SA, separated by approximately 864 kilobases. Within each GWAS, SNPs show high LD amongst themselves (D′ ≈ 1.0, *R* ^2^ ≈ 1.0), indicating a strong correlation. However, SNPs between the two GWAS are less correlated; for instance, rs1977199 shows moderate LD (*R* ^2^ = 0.2281) with the top SNP in the thalamus GWAS.

Given the complexity of the MHC region and the observed LD patterns, we conducted further analyses to determine whether the shared genomic segments represent independent genetic signals or a single shared variant influenced by the MHC's LD structure.

### 4.3 Conditional and LD Analyses of Shared Signals in the MHC Region

To investigate whether the seven shared genomic segments between SA and thalamus RBV in the MHC region represent independent genetic signals, we performed COJO and LD analyses. The COJO analysis identified distinct lead SNPs for SA (rs34816374 at position 26,949,672 bp) and thalamus volume (rs9295687 at position 26,082,710 bp) on chromosome 6.

The LD analysis between these lead SNPs revealed a low *R* ^2^ value of 0.04, indicating low correlation and suggesting that the SNPs are not in strong LD. Despite a high D' value of 0.89, which indicates limited historical recombination, the low *R* ^2^ implies that the SNPs do not predict each other's alleles well due to differences in allele frequencies.

In the conditional analysis, we found that the association of rs34816374 with SA remained genome‐wide significant after conditioning on rs9295687 (*p* = 4.04 × 10^−8^). Similarly, the association of rs9295687 with thalamus RBV remained significant, although with a slightly higher *p* value (*p* = 1.21 × 10^−6^) after conditioning on rs34816374. These results suggest that the associations are likely due to independent genetic signals rather than a single shared variant.

**FIGURE 3:**
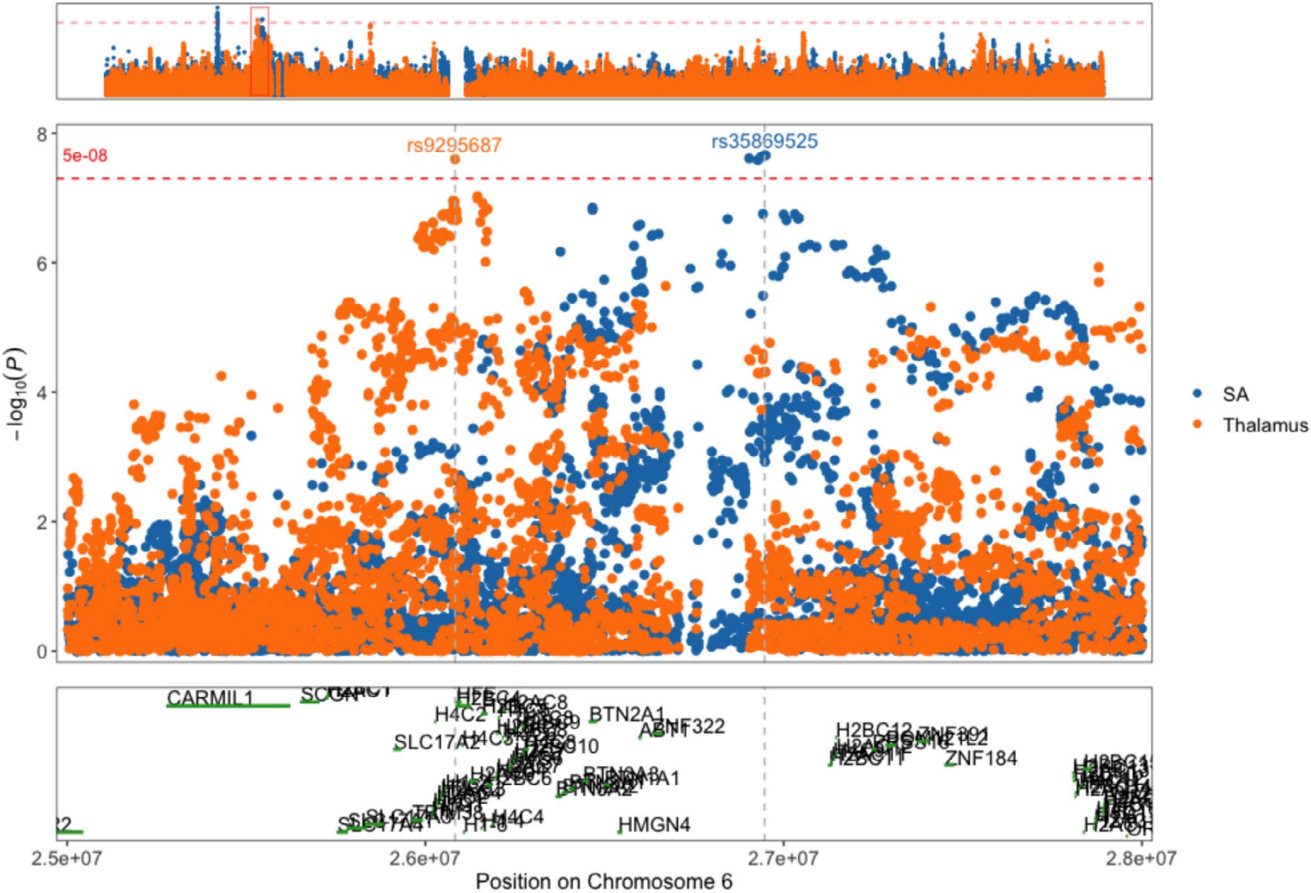
Regional association plots for suicide attempt (SA) and thalamus volume in the MHC region on chromosome 6.

Figure 3 illustrates the regional association plots for SA and thalamus RBV in the MHC region. It highlights the lead SNPs and demonstrates the distinct association peaks for each trait.

### 4.4 PGS Associations in ABCD

Table 2 displays the results of SA PGS predicting ICV and subcortical RBVs. Before FDR correction, higher SA PGS was associated with smaller ICV (*b* = −8337.2, *p* = 0.007), smaller RBVs of the brainstem (*b* = −87.97, *p* = 0.012) and right nucleus accumbens (*b* = −7.05, *p* = 0.001). After FDR adjustment, only the association with the SA PGS and right nucleus accumbens remained significant (*p* = 0.018).

**TABLE 2:** Suicide attempt polygenic risk score associations with intracranial volume and subcortical brain structure phenotypes.

Outcome	*b*	SE	Unadjusted *p*	FDR‐adjusted *p*
ICV	−8337.2	3065.25	**0.007** **	0.060
Right caudate	−11.88	10.20	0.244	0.437
Left caudate	−10.00	10.22	0.328	0.437
Right putamen	9.10	11.92	0.446	0.472
Left putamen	7.26	12.36	0.557	0.557
Right hippocampus	−10.66	8.35	0.202	0.437
Left hippocampus	−7.37	7.72	0.340	0.437
Brainstem	−87.97	34.96	**0.012** *	0.072
Right ventral diencephalon	−10.61	7.00	0.130	0.342
Left ventral diencephalon	−12.67	7.05	0.073	0.263
Right thalamus	11.99	12.44	0.336	0.437
Left thalamus	13.95	14.22	0.327	0.437
Right global pallidus	5.54	4.95	0.264	0.437
Left global pallidus	4.38	5.40	0.417	0.469
Right nucleus accumbens	−7.05	2.19	**0.001** **	**0.018** *
Left nucleus accumbens	4.38	5.40	0.417	0.469
Right amygdala	−6.69	4.44	0.133	0.342
Left amygdala	−8.84	4.85	0.069	0.263

*Note:* Bold indicates statistical significance.

Abbreviations: FDR = false discovery rate; ICV = intracranial volume; SE = standard error.

* *p* < 0.05.

** *p* < 0.01.

## 5 Discussion

This study explored the genetic overlap between SA and RBVs by leveraging findings from large‐scale GWAS on these traits. We found a significant negative genetic correlation between SA and ICV, suggesting that genetic factors associated with increased SA risk are linked to smaller brain size. These findings align with previous research indicating that individuals who have attempted suicide exhibit smaller ICV compared to healthy controls (Cohen's d = −0.13) (Campos et al. 2021). Our results are also consistent with findings from another study that observed smaller ICV in patients with major depressive disorder who made suicidal plans or attempts (Cohen's d = −0.284) compared to healthy controls (Rentería et al. 2017). These findings indicate a potential shared genetic or biological basis between SA and RBVs, though the directionality or causality of this relationship remains uncertain and requires further investigation.

Using local genetic overlap analysis with GWAS‐PW, we identified 10 genomic segments shared between SA and RBVs, notably the thalamus, putamen and caudate nucleus. The RBV of the thalamus showed the largest number of shared genomic segments (*N* = 7) with SA, highlighting its potential role in SA's genetic architecture. The thalamus is crucial for sensory and motor signal relay and regulating consciousness, sleep and alertness (Torrico and Munakomi 2023). Genetic factors influencing thalamic morphology may affect emotional regulation and stress response (Hsu et al. 2014), which are critical in SA.

Our conditional and linkage disequilibrium analyses in the MHC region indicated that the shared associations between SA and thalamus RBV are due to independent genetic signals rather than a single variant. This finding strengthens the evidence for the thalamus's involvement in SA and suggests distinct biological pathways warrant further investigation. Functional annotation revealed several genes significantly associated with both SA and at least one of three subcortical RBVs (thalamus, putamen and caudate nucleus). We observed seven genes associated with the RBV of the thalamus (*BTN3A2*, *HIST1H1B*, *HIST1H2BL*, *HIST1H2BN*, *HIST1H2AJ*, *HIST1H4L* and *OR2B2*), followed by two with the RBV of the putamen (*PPP4R1* and *DCC*) and one with the RBV of the caudate nucleus (*DCC*). In particular, *DCC* has been previously associated with SA (Mullins et al. 2022). *DCC* may point to the repatterning of connections in the pyramidal neurons in the MDD brain due to the *DCC*'s ability to control axon arborisation, dendritic growth and synapse formation (Roy and Dwivedi 2018; Torres‐Berrío et al. 2017). Identifying these genes could serve as testable hypotheses for potential targets in future studies seeking to understand the underlying genetic mechanisms of SA.

The putamen and caudate nucleus are integral parts of the basal ganglia (García‐Marín et al. 2023), which are associated with a variety of functions, including motor control, procedural learning and emotional regulation (Lanciego et al. 2012; Ghandili and Munakomi 2023). Dysfunctions in these structures have been linked to mood disorders (Talati et al. 2022; Krishnan et al. 1992), including depression, which is a significant risk factor for SA (Li et al. 2022a, 2022b). The genetic overlap between SA and these structures implies that shared genetic factors may contribute to neurobiological pathways that influence brain morphometry and SA risk.

We identified three genes (*BTN3A2*, *HIST1H2BN* and *HIST1H4L*) that are associated with both SA and major psychiatric disorders, such as MDD (Yang et al. 2021), schizophrenia (SCZ) (Li et al. 2022a, 2022b), bipolar disorder and autism spectrum disorder (Yulu Wu et al. 2020), suggesting a potential genetic overlap and pleiotropic effects across these conditions. The *BTN3A2* gene has been linked to immune dysregulation response from the extended major histocompatibility complex (xMHC) region in MDD and SCZ (Tubbs et al. 2020; Yong Wu et al. 2019; Yang et al. 2021). Furthermore, we observed associations with the *HIST1H2BN* and *HIST1H4L* genes, which have been linked previously to SCZ, MDD, bipolar disorder and autism spectrum disorder (Yulu Wu et al. 2020). These findings align with previous associations reported on mental health disorders and might be part of the pleiotropy of SA with major psychopathology (DiBlasi et al. 2021). Besides, histonic methylation from histonic genes (*HIST1H1B*, *HIST1H2BL*, *HIST1H2BN*, *HIST1H2AJ* and *HIST1H4L*) plays a prominent role in bipolar disorder and synapse and postsynapse‐related processes of SCZ (Network and Pathway Analysis Subgroup of Psychiatric Genomics Consortium 2015). The genes associated with these independent signals, such as the HIST1H family genes and BTN3A2, may have different functional roles in neurodevelopment or immune‐related processes. Our results emphasise the importance of considering the complexity of genomic regions like the MHC when interpreting GWAS findings and highlight the utility of conditional and LD analyses in disentangling overlapping association signals.

In terms of the olfactory receptor gene (*OR2B2*) this gene has been linked to Alzheimer's disease in a Japanese cohort (Shigemizu et al. 2022). The involvement of genes related to neurodevelopment and neuroplasticity, such as *DCC* and the histone cluster genes, indicates that dysregulation in these pathways can lead to structural and functional brain abnormalities, increasing the risk of SA. The broader implications of *OR2B2* in brain function and neural connectivity underscore the complex interplay between sensory processing, neural circuit formation and psychiatric health, which are crucial for emotional and cognitive regulation. Lastly, the protein phosphatase 4 regulatory subunit 1 (*PPP4R1*) has been reported to be involved in cancer development, especially with poor prognosis, drug resistance, and natural killer cell‐mediated toxicity (Xiaowei Li et al. 2024). These associations might also be implicated in pathways associated with SA, which is consistent with work that SA is often a reflection of the complex interplay between genetic predisposition and multiple biological processes (Mullins et al. 2022).

In the ABCD cohort, higher PGS for SA was associated with a smaller volume of the right nucleus accumbens after correction for multiple testing. The nucleus accumbens is involved in reward processing and emotional regulation (Levita et al. 2009; Klawonn and Malenka 2018). Adolescents differ markedly from adults in terms of neurodevelopmental trajectories, impulsivity and emotional reactivity (Casey et al. 2008; Dumontheil 2016). For example, previous research has shown that subcortical structures, including the amygdala and nucleus accumbens, mature earlier in development compared to cortical regions such as the prefrontal cortex, which is thought to continue developing into young adulthood. In line with dual systems models of development, maturational imbalances between brain systems linked to reward processing compared to those associated with cognitive control are thought to underlie poorer self‐regulatory capacities and risk‐taking behaviours that characterise adolescence (Mills et al. 2014). Our findings suggest that genetic predispositions associated with SA may manifest distinctly during adolescence, possibly through alterations in neural systems implicated in reward, risk‐taking and affective dysregulation. This observation differs from those previously reported for adult suicide, which often is preceded by different psychosocial stressors, chronic psychiatric disorders and distinct neural pathways shaped by prolonged exposure to environmental factors. Therefore, the observed structural brain differences linked to genetic risk for SA in adolescence may uniquely reflect developmental vulnerabilities rather than reflect neural phenotypes associated with suicidal behaviour in adult populations. Further research is needed to determine whether the association between genetic risk for SA is linked to a smaller volume of the right nucleus accumbens in adult samples.

We observed that the application of different analytical approaches revealed distinct brain structures implicated in SA. For example, whilst genetic correlation analyses (LDSC) revealed a significant relationship between SA and ICV, pairwise GWAS analyses identified genomic segments overlapping between SA and subcortical volumes (e.g., thalamus, putamen and caudate nucleus). Functional annotation of these overlapping genomic segments revealed their biological relevance through enriched pathways potentially implicated in psychopathology. However, in PGS analyses within the ABCD cohort, we observed that the SA PGS was significantly associated with smaller volume of the right nucleus accumbens. The divergence of findings between GWAS‐PW and PGS likely reflects the application of distinct methodologies and sample characteristics. For instance, GWAS‐PW analyses captured overlapping genetic variants between traits in adult cohorts, potentially indicating broader genomic associations. In contrast, the PGS analyses focused on the cumulative impact of genetic variants within a younger, neurodevelopmentally distinct adolescent sample, where the genetic vulnerability may preferentially manifest in brain regions involved in reward and emotional processing, such as the nucleus accumbens (Mills et al. 2014). Thus, whilst several brain structures appear to be genetically linked to SA across analyses, the nucleus accumbens may uniquely represent a neurodevelopmentally sensitive structure reflecting adolescent‐specific vulnerabilities. Future studies integrating longitudinal data and cross‐age analyses will be essential to clarify these developmental trajectories and the reasons underlying methodological divergence.

Our study provides important insights into the study of SA neurogenetics. Our results are consistent with recent evidence of genes associated with SA that are highly expressed in the brain and are involved in various biological processes, including epigenetics, gene regulation and transcription, cellular stress responses, DNA repair mechanisms and immunological signatures (Docherty et al. 2023), which have been previously associated with SA within the hypothalamic–pituitary–adrenal (HPA) system dysregulation (Pfennig et al. 2005). This evidence might confirm the hypothesis that neurobiological differences might lead individuals to higher SA risk.

However, it is essential to note that our findings related to the genetic association analyses were conducted at the local and genome‐wide levels; therefore, they do not imply causality. Whilst shared genetic liability between SA and brain morphology is likely, given the high polygenicity of these phenotypes, it remains possible that genetic factors could influence one phenotype and indirectly affect the other through a causal pathway. Future studies can explore this possibility by applying methods such as two‐sample Mendelian Randomisation (MR) to evaluate support for causal relationships between brain morphology and SA. However, such analyses are beyond the scope of the present study, which focuses on shared genetic associations. Also, our research only included participants of European ancestry, so our results should be considered only representative of white European ancestry individuals until confirmed in other ancestries. Moreover, whilst our analyses included multiple subcortical structures, we did not examine cortical regions previously associated with suicidal behaviors, such as the ventrolateral and dorsolateral prefrontal cortices (Schmaal et al. 2020). Future studies should explore genetic associations between SA and cortical brain structures, which may allow for a more comprehensive mapping of neural structural correlates associated with genetic risk for SA.

Further research using larger sample sizes and including participants from diverse ancestral backgrounds is needed to elucidate these potential causal relationships. These findings open new avenues for exploring how genetic factors contribute to the risk of SA, particularly through mechanisms related to immune response, oxidative stress and chronic inflammation. In addition, our results highlight specificity in the association between PGS for SA and RBV of the right nucleus accumbens in adolescents, underscoring its potential as an endophenotype through which genetic risk for SA may manifest early in development. The nucleus accumbens is a key structure involved in reward processing, motivation and emotional regulation, all of which are critical to understanding the neurobiological underpinnings of SA.

Future work should focus on elucidating the specific molecular pathways linking genetic variants associated with SA to neuropsychiatric outcomes, which could ultimately lead to identifying novel therapeutic targets for preventing SA. Integrating genetic and neuroimaging data in the context of SA holds promise for developing more effective prediction models (van Velzen et al. 2022; Campos et al. 2021). By enhancing our understanding of the shared genetic factors influencing SA and brain morphology, this approach could significantly improve our capacity to identify individuals at increased risk across the lifespan. Whilst challenges remain in scaling these methods for clinical application, ongoing technological advancements may facilitate their integration into preventive strategies.

## Author Contributions

All authors made a significant contribution to the work reported, whether that is in the conception, study design, execution, acquisition of data, analysis and interpretation, or all these areas; took part in drafting, revising or critically reviewing the article; gave final approval of the version to be published; agreed on the journal to which the article has been submitted and agreed to be accountable for all aspects of the work.

## Conflicts of Interest

The authors declare no conflicts of interest.

## Data Availability

GWAS summary statistics from the ENIGMA‐CHARGE consortium were obtained via a data application. GWAS summary statistics from The International Suicide Genetics Consortium (ISGC) were obtained via direct application and data transfer agreement approval.
